# Intrinsic cardiac adrenergic cells contribute to LPS-induced myocardial dysfunction

**DOI:** 10.1038/s42003-022-03007-6

**Published:** 2022-01-25

**Authors:** Duomeng Yang, Xiaomeng Dai, Yun Xing, Xiangxu Tang, Guang Yang, Andrew G. Harrison, Jason Cahoon, Hongmei Li, Xiuxiu Lv, Xiaohui Yu, Penghua Wang, Huadong Wang

**Affiliations:** 1 Department of Pathophysiology, Key Laboratory of State Administration of Traditional Chinese Medicine of the People’s Republic of China, School of Medicine, Jinan University, Guangzhou, 510632 Guangdong China; 2 Department of Pathogen biology, School of Medicine, Jinan University, Guangzhou, 510632 Guangdong China; 3 Department of Immunology, University of Connecticut Health Center, 263 Farmington Ave., Farmington, CT 06030 USA

**Keywords:** Cardiomyopathies, Sepsis, Toll-like receptors, Signal transduction, Sepsis

## Abstract

Intrinsic cardiac adrenergic (ICA) cells regulate both developing and adult cardiac physiological and pathological processes. However, the role of ICA cells in septic cardiomyopathy is unknown. Here we show that norepinephrine (NE) secretion from ICA cells is increased through activation of Toll-like receptor 4 (TLR4) to aggravate myocardial TNF-α production and dysfunction by lipopolysaccharide (LPS). In ICA cells, LPS activated TLR4-MyD88/TRIF-AP-1 signaling that promoted NE biosynthesis through expression of tyrosine hydroxylase, but did not trigger TNF-α production due to impairment of p65 translocation. In a co-culture consisting of LPS-treated ICA cells and cardiomyocytes, the upregulation and secretion of NE from ICA cells activated cardiomyocyte β_1_-adrenergic receptor driving Ca^2+^/calmodulin-dependent protein kinase II (CaMKII) to crosstalk with NF-κB and mitogen-activated protein kinase pathways. Importantly, blockade of ICA cell-derived NE prevented LPS-induced myocardial dysfunction. Our findings suggest that ICA cells may be a potential therapeutic target for septic cardiomyopathy.

## Abstract (web summary)

Yang et al report that intrinsic cardiac adrenergic cell-derived norepinephrine promotes myocardial dysfunction in response to LPS. They demonstrate norepinephrine produced by these cells activated β1-adrenergic receptors on cardiomyocytes increasing the expression of tumor necrosis factor-a in a Ca^2+^/calmodulin-dependent protein kinase II dependent manner.

## Introduction

Myocardial dysfunction is a frequent event that correlates with the severity of sepsis, which accounts for the primary cause of death in intensive care units^1,2^. During sepsis-induced myocardial dysfunction (SIMD), pathogen-associated molecular patterns (PAMPs), such as bacterial lipopolysaccharide (LPS), interact with Toll-like receptor 4 (TLR4) on immune and cardiac cells, activating NF-κB and mitogen-activated protein kinase (MAPK) signaling cascades to produce proinflammatory cytokines, including TNF-α, interleukin-1β and interleukin-6. These cytokines act on endothelial cells, cardiac fibroblasts, and cardiomyocytes to increase the production of inflammatory mediators, which cause myocardial depression^3–8^. Norepinephrine (NE), demonstrated to be associated with SIMD^9,10^, can activate β_1_-adrenergic receptor (AR) and promote Ca^2+^/calmodulin-dependent protein kinase II (CaMKII) phosphorylation, IκBα phosphorylation, and TNF-α expression, all of which contribute to cardiomyocyte apoptosis in septic mice^11^. Moreover, selective β_1_-AR blockade improves cardiac function and survival in septic patients^12,13^.

The intrinsic cardiac adrenergic (ICA) cells, which express tyrosine hydroxylase (TH) and dopamine-β-hydroxylase (DBH) necessary for catecholamine biosynthesis, have been identified in the mammalian heart as an important origin of intrinsic cardiac catecholamines^14,15^. A growing body of literature has suggested that the ICA system is a critical regulator of mammalian heart development, post-heart transplantation inotropic support, and cardioprotection during ischemia/reperfusion^15–21^. However, the response of ICA cells in SIMD is unclear. Our previous study showed that blockade of β_1_-AR suppressed LPS-induced TNF-α production in primary neonatal rat cardiomyocytes cultured by traditional methods in the absence of exogenous NE^22^, indicating that some β_1_-AR stimuli function in the culture. In addition, ICA cells were found to be present in the primary neonatal rat cardiomyocyte culture using traditional methods^14,23^. In this context, we postulated that LPS might stimulate ICA cells to synthesize NE that would promote LPS-induced cardiomyocyte TNF-α production and myocardial dysfunction via β_1_-AR.

In this study, we investigate the effect of LPS on NE production in ICA cells and the role of ICA cell-derived NE in LPS-induced myocardial dysfunction. We demonstrate that LPS increases NE production in ICA cells that express TLR4, and identify ICA cell-derived NE as a paracrine signal to enhance LPS-provoked proinflammatory response in cardiomyocytes and myocardial dysfunction via β1-AR-CaMKII-NF-κB/MAPKs signaling cascades.

## Results

### ICA cells are present in neonatal and adult rat hearts

**Fig. 1:**
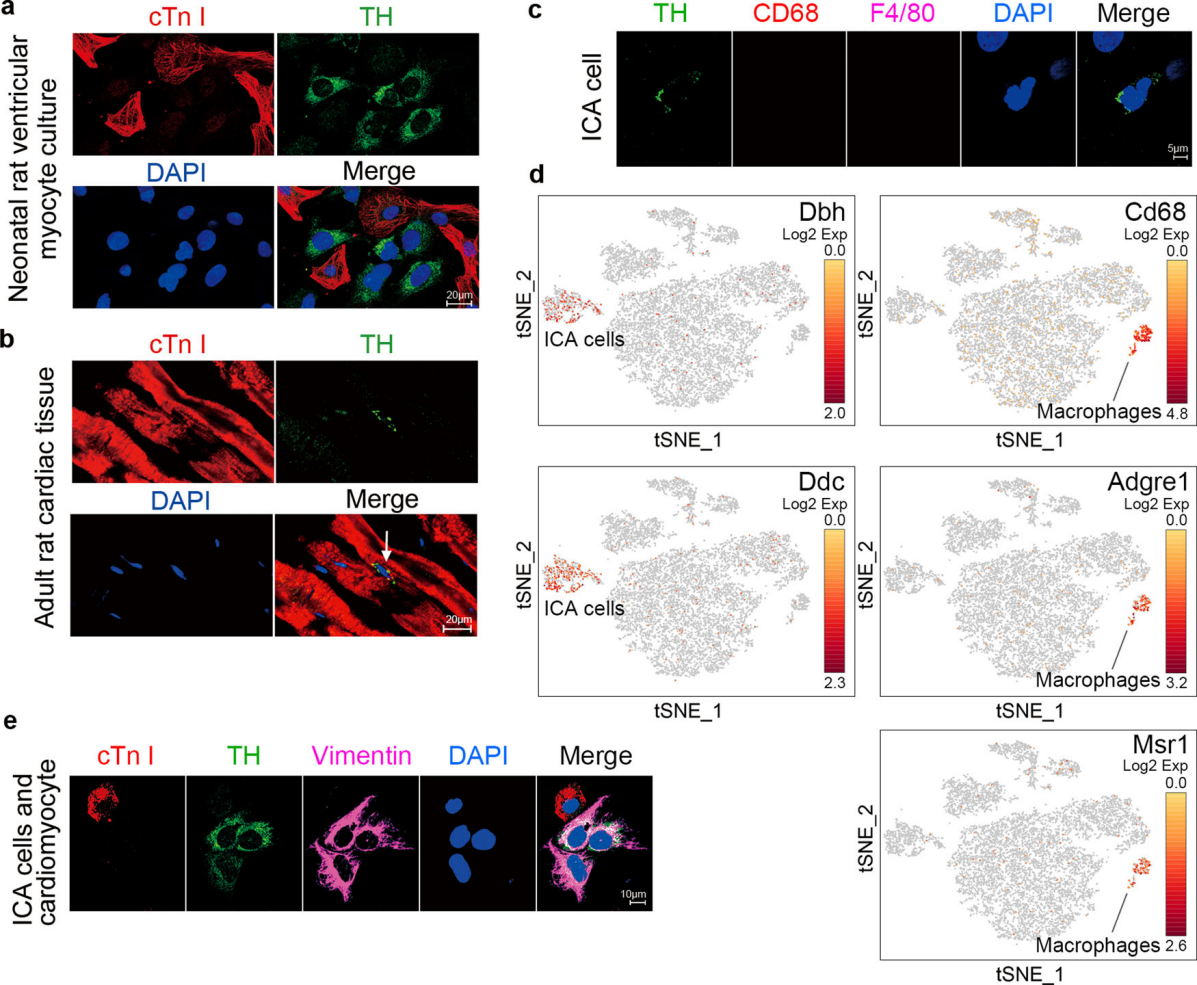
Intrinsic cardiac adrenergic (ICA) cells are present in neonatal and adult rat hearts. **a** Immunofluorescent staining of primary cardiomyocytes and ICA cells isolated from neonatal rat hearts using traditional method (*n* = 6). **b** Immunofluorescent staining of adult rat cardiac tissue slides (*n* = 3). cTn I (cardiac troponin I): cardiomyocytes, red; TH (tyrosine hydroxylase): ICA cells, green (white arrow). **c** ICA cells identified using macrophage markers, TH: ICA cell, green; CD68: marker of macrophage, red; F4/80: marker of macrophage, magenta (*n* = 6). **d** Single-cell RNA-sequencing analysis of cardiac cells obtained using the Percol method. Note, both ICA cells and macrophages are identified as two different clusters. *Dbh* codes Dopamine Beta-Hydroxylase, *Ddc* codes Dopa Decarboxylase, *Cd68* codes CD68, *Adgre1* codes F4/80, and *Msr1* codes MSR1 (*n* = 24). **e** Immunostaining of ICA cells and cardiomyocyte, cTn I: cardiomyocyte, red; TH: ICA cells, green; Vimentin: marker of mesenchymal cells, magenta (*n* = 6). Nuclei of cells in all groups were dyed using DAPI: blue. The data represent two independent experiments, *n* represents the number of neonatal rats (**a**, **c**, and **d**) or adult rats (**b**).

Immunoreactivity of TH, a key catecholamine-forming enzyme, in ICA cells has been demonstrated previously (Fig. S1B)^19^, and the established 3D structure of ICA cells illustrated its TH immunoreactivity (Fig. S1C). We thus performed immunofluorescent staining of TH to identify ICA cells. As shown in Fig. 1a, in the culture of cardiomyocytes isolated from neonatal rat hearts using a traditional enzymatic method^24^, the TH-positive ICA cells were found to cluster nearby cardiac troponin I (cTn I)-positive neonatal rat ventricular myocytes (NRVM). In addition, these ICA cells were also found in adult rat hearts by staining of TH and cTn I in tissue slices (Fig. 1b, white arrow). Macrophages were reported as a source of catecholamines in response to acute inflammatory injury^25^, but we demonstrated that ICA cells did not express macrophage markers CD68 or F4/80 (Fig. 1c and Fig. S1D, E). Moreover, single-cell RNA sequencing revealed that ICA cells (present *Dbh* and *Ddc* coding for Dopa Decarboxylase) and macrophages (present *Cd68*, *Msr1,* and *Adgre1* coding for F4/80) were in two distinct clusters (Fig. 1d). Therefore, ICA cells were not considered to be of the macrophage lineage. Since vimentins are type III intermediate filaments expressed especially in mesenchymal cells, co-staining of vimentin, cTn I, and TH in co-culture of ICA cells and cardiomyocytes suggested that ICA cells are of mesenchymal cell origin (Fig. 1e). To validate the specificity of each staining, negative controls are provided in Fig. S2.

### Increased NE release from ICA cells promotes cardiomyocyte TNF-α production in response to LPS challenge

**Fig. 2:**
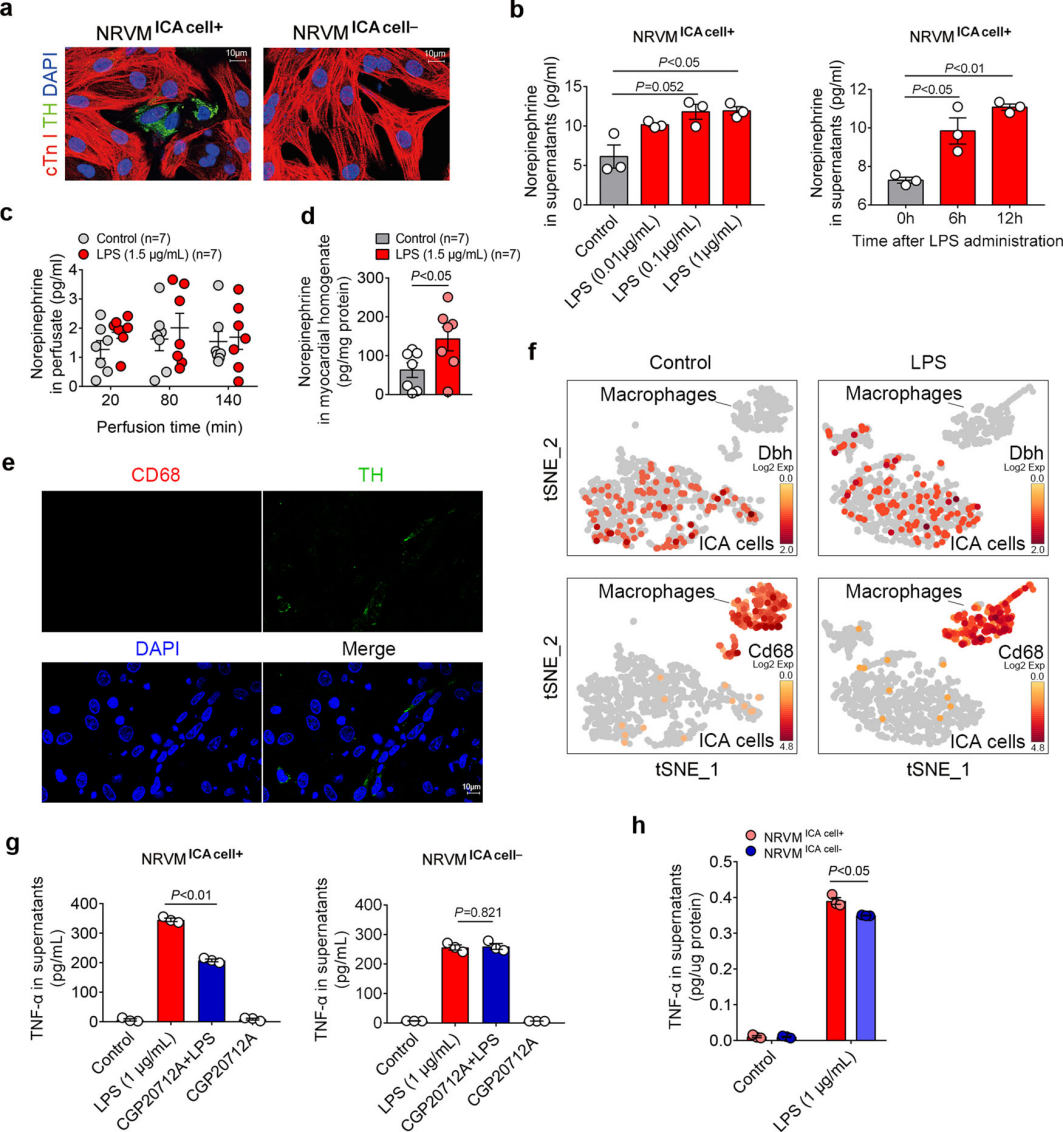
LPS-induced ICA cell-derived NE enhances cardiac TNF-α production. **a** Immunofluorescent staining of neonatal rat ventricular myocyte (NRVM) co-culturing with ICA cells (NRVM ^ICA cell+^) and NRVM without ICA cells (NRVM ^ICA cell−^), cTn I: cardiomyocytes, red; TH: ICA cells, green; DAPI: nuclei, blue (*n* = 12). **b** Norepinephrine (NE) level in supernatants of NRVM ^ICA cell+^ stimulated with different doses of LPS for 6 h, and with 1 µg/mL LPS for different durations (*n* = 36, each dot represents one independent repeat). **c** NE in perfusate from Langendorff-perfused adult rat hearts, control: K-H buffer. **d** NE in myocardial homogenates from adult rat hearts Langendorff perfused for 140 min, control: K-H buffer (*n* = 7 rats per group in **c** and **d**, each dot represents an adult rat). **e** Immunostaining of cells in current cardiomyocyte culture, CD68: marker of macrophage, red; TH: maker of ICA cells, green (*n* = 6). **f** Single-cell RNA-sequencing analysis of cardiac cells obtained using the Percol method, containing both ICA cells and macrophages treated with LPS. *Dbh* codes Dopamine Beta-Hydroxylase, *Cd68* codes CD68 (*n* = 24). **g** TNF-α in supernatants 6 h post LPS-treated NRVM ^ICA cell+^ and NRVM ^ICA cell-^ pretreated with 2 µM CGP20712A for 30 min prior to LPS. **h** TNF-α in supernatants of NRVM ^ICA cell+^ and NRVM ^ICA cell−^ stimulated with 1 µg/mL LPS for 6 h (*n* = 36, each dot represents one independent repeat in **g** and **h**). Data are presented as mean ± SEM, the *P*-values were assigned on each panel. **b** the LPS dose data were analyzed using non-parametric Kruskal–Wallis test and Dunn’s method of comparison, the duration data and **g** and **h** were analyzed using one-way ANOVA and Bonferroni multiple comparison test; **c** multiple *t-*test followed by the Holm-Sidak method; **d** independent Student’s *t-test*. The in vitro data represent three independent experiments. *n* represents the number of neonatal rats (**a**, **b**, **e**–**h**) or adult rats (**c**, **d**).

The primary NRVM culture containing ICA cells is defined as a co-culture of ICA cell and NRVM (NRVM ^ICA cell+^). To analyze the role of ICA cells, we used an established method of superparamagnetic iron oxide particles (SIOP) to obtain pure NRVM that contains no ICA cells (NRVM ^ICA cell−^) (Fig. 2a)^24^. LPS significantly raised NE levels in the supernatants of NRVM ^ICA cell+^ with either different doses of LPS (LPS 0.01 μg/mL, 0.1 μg/mL, and 1 μg/mL) or different treatment durations (LPS 0, 6, and 12 h) when compared with controls (Fig. 2b). During the Langendorff perfusion of adult rat hearts, LPS elicited negligible influences on NE levels in perfusate, suggesting that circulating NE was not involved due to the lack of sympathetic nerves and the adrenal medulla in the Langendorff model (Fig. 2c). However, LPS administration significantly increased the level of NE in myocardial homogenate (Fig. 2d). Immunostaining confirmed that macrophages were not present in the current cardiomyocyte culture system (Fig. 2e), and single-cell RNA sequencing demonstrated that *Dbh* was not induced in LPS-treated *Cd68* positive macrophages, which was expressed in ICA cells (Fig. 2f). Therefore, the increase of NE level in NRVM ^ICA cell+^ culture conditions and adult rat myocardial homogenate could be attributed to the ICA cells, but not macrophages. Consequently, we found that blockade of β_1_-AR using CGP20712A, a selective β_1_-AR antagonist, dramatically suppressed LPS-induced TNF-α production in NRVM ^ICA cell+^, whilst removal of ICA cells abolished the impact of CGP20712A on TNF-α production (Fig. 2g). Moreover, the removal of ICA cells caused a lower level of TNF-α in the supernatants of LPS-stimulated NRVM ^ICA cell−^ than that of NRVM ^ICA cell+^ (Fig. 2h). These data support the concept that LPS stimulates ICA cell-derived NE release, which promotes LPS-induced TNF-α production in cardiomyocytes.

### LPS upregulates NE-producing enzyme expression in ICA cells

**Fig. 3:**
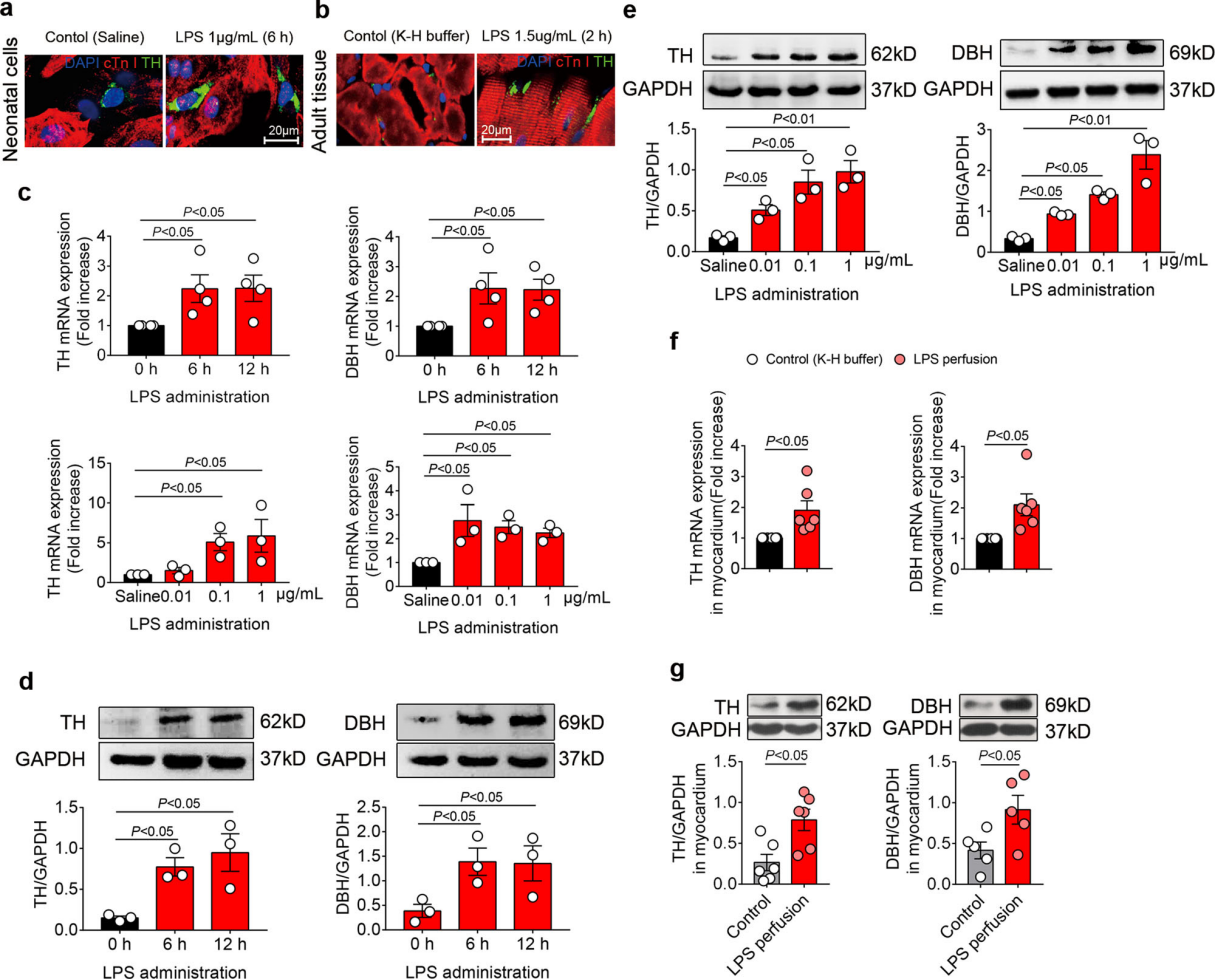
LPS stimulation upregulates NE-producing enzymes in ICA cells. **a** and **b** TH expression in NRVM ^ICA cell+^ treated with saline or LPS for 6 h (**a**, *n* = 6) and adult rat hearts Lagendorff perfused for 2 h (**b**, *n* = 6 per group); cTn I: cardiomyocytes, red; TH: ICA cells, green; DAPI: nuclei, blue. **c**–**e** mRNA and protein expression of TH and DBH relative to GAPDH in NRVM ^ICA cell+^ stimulated with LPS at different time points and doses (*n* = 36, one-way ANOVA and Bonferroni multiple comparison test, each dot represents one independent repeat). **f** and **g** mRNA and protein expression of TH and DBH relative to GAPDH in myocardium from adult rat hearts Lagendorff perfused for 2 h, control: K-H buffer, LPS: 1.5 µg/mL in K-H buffer (*n* = 6 rats per group, independent Student’s *t-test*, each dot represents a rat). Data are presented as mean ± SEM. *P*-values were assigned on each panel. *n* represents the number of neonatal rats (**a**–**e**) or adult rats (**f**–**g**).

We next intended to elucidate how NE release is increased following LPS treatment. Given TH and DBH are the key enzymes responsible for NE biosynthesis^9^, we evaluated the expression of TH and DBH in ICA cells. Immuno-staining showed that LPS significantly stimulated TH expression in neonatal and adult rat ICA cells (Fig. 3a, b). In the NRVM ^ICA cell+^ culture, LPS (1 μg/mL) stimulation for 6 h and 12 h dramatically increased TH and DBH mRNA expression, and different doses of LPS also significantly enhanced TH and DBH mRNA expression after 6 h compared to controls (Fig. 3c). Consistently, a dramatic increase was observed in the protein expression of TH and DPH following LPS treatment (Fig. 3d, e). In Langendorff-perfused adult rat hearts, LPS increased TH and DBH expression significantly at both mRNA and protein levels compared to controls (Fig. 3f, g). These data show that LPS increases NE release by upregulating TH and DBH expression in ICA cells.

### LPS increases TH and DBH biosynthesis through TLR4-MyD88/TRIF-AP-1 signaling

**Fig. 4:**
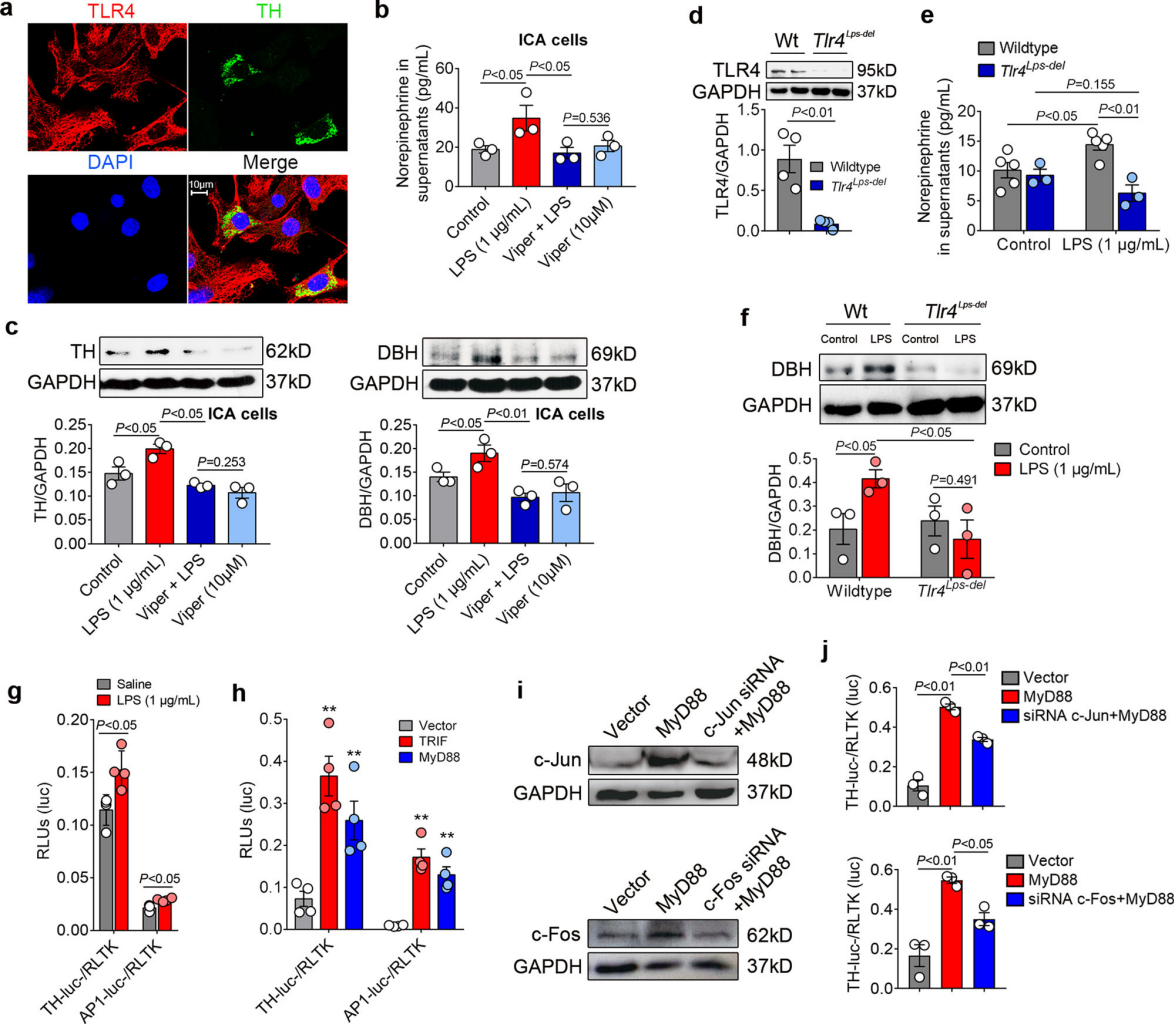
LPS-TLR4/AP-1 signaling mediates NE-producing enzyme expression. **a** Co-immunofluorescent staining of TLR4 and TH in NRVM ^ICA cell+^, TLR4: toll-like receptor 4, red; TH: ICA cells, green; DAPI: nuclei, blue (*n* = 6). **b** and **c** NE production as well as TH and DBH expression in rat ICA cells treated with Viper (TLR4 inhibitor, 10 μM) for 2 h prior to LPS for 6 h. (*n* = 48, each dot represents one independent repeat). **d** TLR4 expression in wildtype (WT) and TLR4-deficient (*Tlr4*^*Lps-del*^) NMVM ^ICA cell+^. **e** and **f** NE production and DBH expression in WT and *Tlr4*^*Lps-del*^ NMVM ^ICA cell+^ stimulated with LPS for 6 h, control: saline (*n* = 12, each dot represents a biological replicate in **d**–**f**). **g** Luciferase activity of AP-1-luc- and TH-promoter-luc- relative to RLTK-luc- in HEK293/hTLR4 cells at 36 h after transfection and LPS stimulation. **h** Luciferase activity of AP-1-luc- and TH-promoter-luc- relative to RLTK-luc- in HEK293/hTLR4 cells at 24 h after transfection with plasmids (0.1 μg/mL) encoding MyD88 and TRIF. **i** Expression of c-Jun and c-Fos in HEK293/hTLR4 cells at 24 h after transfection with MyD88 plasmid, c-Jun siRNA and c-Fos siRNA. **j** Luciferase activity of TH-promoter-luc- relative to RLTK-luc- in HEK293/hTLR4 cells at 24 h after transfection with MyD88 plasmid, c-Jun siRNA, and c-Fos siRNA, (0.1 μg/mL for plasmid) (each dot represents one independent repeat in (**g**–**j**)). Data are presented as mean ± SEM using one-way ANOVA and Bonferroni multiple comparison test, *P < 0.05, **P < 0.01, most of the *P*-values were assigned on the figure. *n* represents the number of neonatal rats (**a**–**c**) or neonatal mice (**d**–**f**).

**Fig. 5:**
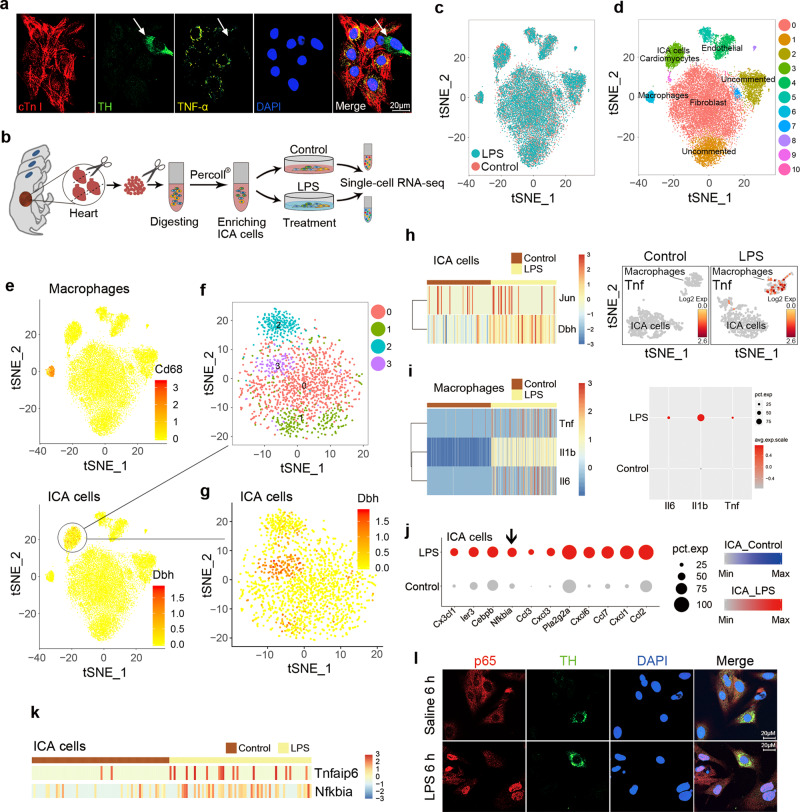
ICA cells do not produce TNF-α upon LPS stimulation. **a** Immunofluorescent-staining of TNF-α in NRVM ^ICA cell+^ stimulated with 1 µg/mL LPS for 6 h, cTn I: cardiomyocytes, red; TH: ICA cell (white arrow), green; TNF-α, yellow; DAPI: nuclei, blue (*n* = 12 neonatal rats). **b** Schematic of single-cell RNA-sequencing experimental strategy. **c** *t*-distributed stochastic neighbor embedding (t-SNE) projection of cardiac cells, batch effect analysis between control and LPS groups, control: saline, 24,210 cells. **d** t-SNE map of cardiac cells, different colored clusters represent distinct populations, 24,210 cells. **e** ICA cells expressing *Dbh* and macrophages expressing *Cd68* are separated in two clusters, 24,210 cells. **f** and **g** Analysis of substructure in ICA and cardiomyocyte cluster indicates subcluster 3 (Purple) is a ICA cell subpopulation, 1765 cells. **h** Expression of *Dbh* and *Jun* in ICA cells between control and LPS groups, and *Tnf* gene expression in ICA cells and macrophages between control and LPS groups, 138 cells. **i** Expression of *Tnf*, *Il6,* and *Il1b* in macrophages between control and LPS groups, 572 cells. **j** Top 11 differentially expressed genes (DEGs) which include *Nfkbia* in the ICA subcluster between control and LPS groups, control: saline, 138 cells. **k** Expression of *Tnfaip6* and *Nfkbia* in ICA cells between control and LPS groups, 138 cells. **l** Immunofluorescent-staining of p65 localization in NRVM ^ICA cell+^ stimulated with saline or 1 µg/mL LPS for 6 h, p65, red; TH: ICA cells, green; DAPI: nuclei, blue (*n* = 6 neonatal rats). For single-cell transcriptomes (**b**–**j**), *n* = 24 neonatal rats. Gene-expression values represent mean of log; differential expression test: Wilcoxon rank sum test; avg_logFC= log (mean(group1)/mean(group2)); adjusted *p*_value: Bonferroni Correction; min.pct ≥10%; avg_logFC ≥ 0.1.

We next attempted to pinpoint the underlying mechanism of NE-producing enzyme upregulation in ICA cells stimulated by LPS. Since TLR4 is a well-known receptor of LPS^26^, we thus hypothesized that TLR4 activation mediates LPS-induced expression of TH and DBH in ICA cells. To this end, we employed immuno-staining to examine the expression of TLR4 in ICA cells. Cardiomyocytes were included as a positive control on which TLR4 expression has been demonstrated^27^. Immuno-staining of TLR4 in NRVM ^ICA cell+^ and isolated ICA cells showed that ICA cells (as well as cardiomyocytes) expressed TLR4 (Fig. 4a and Fig. S4A). Blockade of TLR4 signaling using the Viper peptide (a blocker of TLR4)^28^ markedly suppressed LPS-induced NE production as well as expression of TH and DBH in ICA cells (Fig. 4b, c). Consistently, the DBH expression and NE production were not LPS inducible in TLR4-deficient (*Tlr4*^*Lps-del*^) NMVM ^ICA cell+^, and lower than those in LPS-treated wild-type controls (Fig. 4d–f). Furthermore, we cloned the rat TH promoter into a luciferase reporter (Fig. S4B–F), and found that LPS stimulation markedly activated the TH promoter as well as the AP-1 promoter (Fig. 4g). Similarly, overexpression of the TLR4 adaptors, MyD88 and TRIF, significantly activated both the TH and the AP-1 promoters (Fig. 4h and Fig. S4H). Of note, AP-1 is a heterodimer composed of proteins belonging to the c-Jun and c-Fos families that have specific binding sites on the TH promoter^29^. To see whether disruption of AP-1 binding impacts TH promoter activation, we knocked down c-Jun and c-Fos using the siRNAs in HEK293/hTLR4-HA cells (Fig. 4i, Fig. S5). The activation of the TH promoter in response to MyD88 overexpression was dramatically suppressed by c-Jun siRNA and c-Fos siRNA (Fig. 4j). In addition, single-cell RNA sequencing verified that LPS induced increased *Jun* expression in ICA cells (Fig. 5h). These results reveal LPS induces NE enzyme biosynthesis via TLR4-MyD88/TRIF-AP1, which leads to activation of the TH promoter.

### TLR4-expressing ICA cells do not produce TNF-α upon LPS stimulation

In order to further clarify the role of ICA cells in LPS-induced cardiac TNF-α production, we investigated whether ICA cells are responsible for TNF-α production. To this end, we performed co-immunofluorescence staining of TNF-α, TH, and cTn I in LPS-treated NRVM ^ICA cell+^. Surprisingly, TNF-α was induced in cardiomyocytes but not in ICA cells (Fig. 5a). To validate this phenotype, we implored single-cell RNA sequencing to confirm TNF-α expression by cardiomyocytes and ICA cells. The batch-to-batch variations between the control and LPS groups were normalized to be comparable (Fig. 5c). Distinct populations of cardiac cells were identified in different clusters on the *t*-SNE map (Fig. 5d). Unsupervised clustering revealed macrophages expressing *Cd68* and ICA cells expressing *Dbh* in two separate clusters, supporting our immuno-staining results that ICA cells were not of monocyte origin (Fig. 5d, e). Analysis of substructure in cluster 3 (ICA cells and cardiomyocytes) showed four distinct groups in which the new sub-cluster 3 (purple) was ICA cells expressing *Dbh* (Fig. 5f, g). Further, differentially expressed genes (DEGs) heat maps and clusters displayed an upregulation of *Dbh* and *Jun* expression, but not *Tnf* expression in LPS-treated ICA cells (Fig. 5h). LPS-stimulated macrophages were used as a positive control, resulting in upregulation of *Tnf*, *Il1b,* and *Il6* gene expression (Fig. 5i). Mechanistically, analyses of DEGs showed significantly increased expressions of *Nfkbia* and *Tnfaip6* in LPS-treated ICA cells compared with controls (Fig. 5j, k and Fig. S6C, D). These data suggest the lack of TNF-α production in ICA cells may be attributed to the upregulation of *Nfkbia* and *Tnfaip6*, which are involved in p65 nuclear translocation^30^, and suppression of NF-κB activation^31^, respectively. We, therefore, performed immunofluorescence staining of p65 localization to examine NF-κB activation in LPS-treated NRVM ^ICA cell+^. Strikingly, nuclear translocation of p65 was significantly induced by LPS in non-ICA cells but not in ICA cells, compared to saline-treated controls (Fig. 5l). These findings suggest that ICA cells lack the capacity of producing TNF-α due to impaired nuclear translocation of p65 by elevation of *Nfkbia* and *Tnfaip6*.

### ICA cell-derived NE acts via β_1_-AR-CaMKII signaling to regulate NF-κB and MAPK pathways in cardiomyocyte

**Fig. 6:**
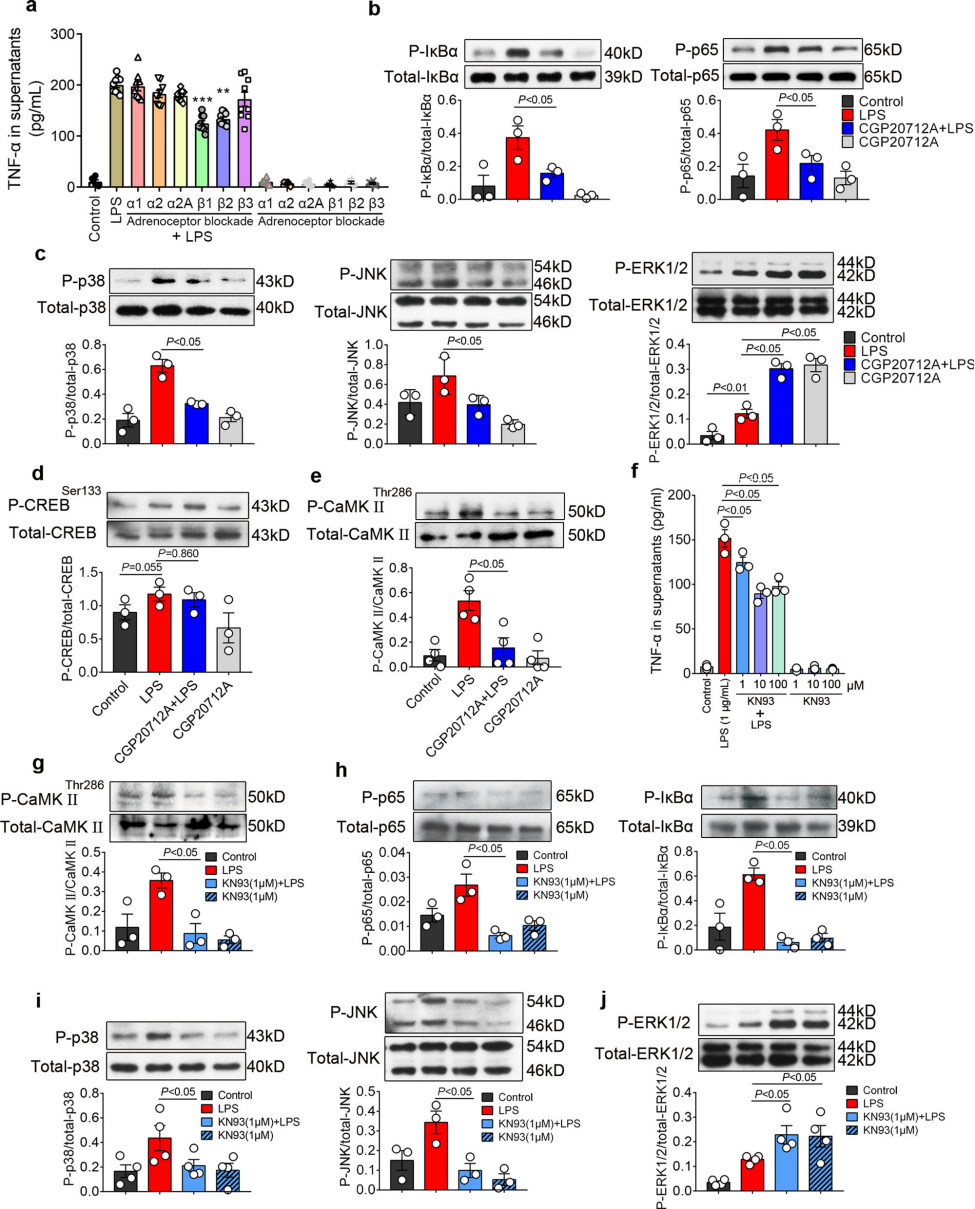
ICA cell-derived NE enhanced by LPS acts via β_1_-AR-CaMKII pathway to regulate NF-κB and MAPK signaling pathways. **a** Effect of different adrenoceptor blockers on TNF-α production in NRVM ^ICA cell+^ upon LPS stimulation. Each dot represents a biological replicate. **b**–**e** NRVM ^ICA cell+^ were stimulated with 2 µM CGP20712A (β_1_-AR antagonist) for 30 min prior to 1 µg/mL LPS for 30 min, **b** phosphorylation of IκBα and p65, **c** phosphorylation of p38, JNK, and ERK1/2, **d** phosphorylation of CREB-Ser133, indicating PKA activation. **e** phosphorylation of CaMKII in NRVM ^ICA cell+^. **f** TNF-α in supernatants of NRVM ^ICA cell+^ treated with KN93 in different doses for 30 min prior to 1 µg/mL LPS for 6 h. **g**–**j** Phosphorylation of CaMKII, p65, IκBα, p38, JNK and ERK1/2 in NRVM ^ICA cell+^ treated with 1 µM KN93 for 30 min prior to 1 µg/mL LPS for 30 min. Data are presented as mean ± SEM using one-way ANOVA and Bonferroni multiple comparison test, *P*-values were assigned on each panel. *n* = 36 neonatal rats (**a**–**j**), data represent three or four independent experiments.

Catecholamines activate various adrenoceptors, to screen out the targets of ICA cell-derived NE in cardiomyocytes, different adrenoceptor inhibitors were employed, in which only β_1_-AR or β_2_-AR blockade significantly reduced the LPS-induced TNF-α production in NRVM ^ICA cell+^ (Fig. 6a). Administration of CGP20712A (2 µM), a β_1_-AR blocker, prior to LPS (1 μg/mL) treatment dramatically suppressed LPS-induced phosphorylation of IκBa and p65 (Fig. 6b), which is consistent with previous work demonstrating activation of β_1_-AR increases IκBα phosphorylation and TNF-α expression in septic mouse hearts^11^. The levels of phosphorylated-p38 and phosphorylated-JNK in the CGP20712A + LPS group were also significantly decreased compared to the LPS alone group, while the ERK1/2 phosphorylation was increased by CGP20712A with or without LPS (Fig. 6c). These results suggest that the NF-κB and MAPK signaling pathways are involved in the process of ICA cell-derived NE promotion of cardiomyocyte TNF-α production. Since protein kinase A (PKA) signaling has proven to be a major route for channeling cardiac β_1_-AR signaling^32^, we thus asked if PKA mediates induction of ICA cell-derived NE on NF-κB and MAPK pathway activation. Intriguingly, the phosphorylation of CREB-Ser133, which indicates PKA activation^33^, showed no significant alteration in either of the groups (Fig. 6d). As the other major downstream element of the β_1_-AR pathway, CaMKII was reported to mediate effects of β_1_-AR stimulation independent of PKA in heart failure, cardiac contractility, and apoptosis^34^. A recent study also showed that CaMKII plays an important role in cardiac contractile dysfunction associated with sepsis^35^. We, therefore, examined the activation of CaMKII in this process. Indeed, the phosphorylation of CaMKII^Thr286^ was dramatically enhanced in LPS-stimulated NRVM ^ICA cell+^ compared with controls, while CGP20712A significantly reduced CaMKII^Thr286^ phosphorylation compared to the LPS group (Fig. 6e). These data hint that CaMKII^Thr286^ phosphorylation is critical in mediating the effects of β-AR stimulation by ICA cell-derived NE. We next used KN93, a selective inhibitor of CaMKII, to block CaMKII signaling. KN93 markedly decreased the levels of TNF-α in the supernatants of LPS-treated NRVM ^ICA cell+^ in a dose-dependent manner (Fig. 6f). The phosphorylation of CaMKII^Thr286^ in the KN93 + LPS group was significantly reduced compared with LPS-treated NRVM ^ICA cell+^ (Fig. 6g). Accordingly, KN93 dramatically decreased LPS-induced phosphorylation of p65, IkBa, p38, and JNK in NRVM ^ICA cell+^ (Fig. 6h, i), and increased ERK1/2 phosphorylation (Fig, 6j). These results show that CaMKII blockade phenocopies the effects of β_1_-AR blockade on LPS-treated NRVM ^ICA cell+^, which suggests that CaMKII is essential during β_1_-AR signal transduction activated by ICA cell-derived NE.

### Dobutamine recapitulates the contributions of ICA cells to cardiomyocyte TNF-α production upon LPS stimulation

**Fig. 7:**
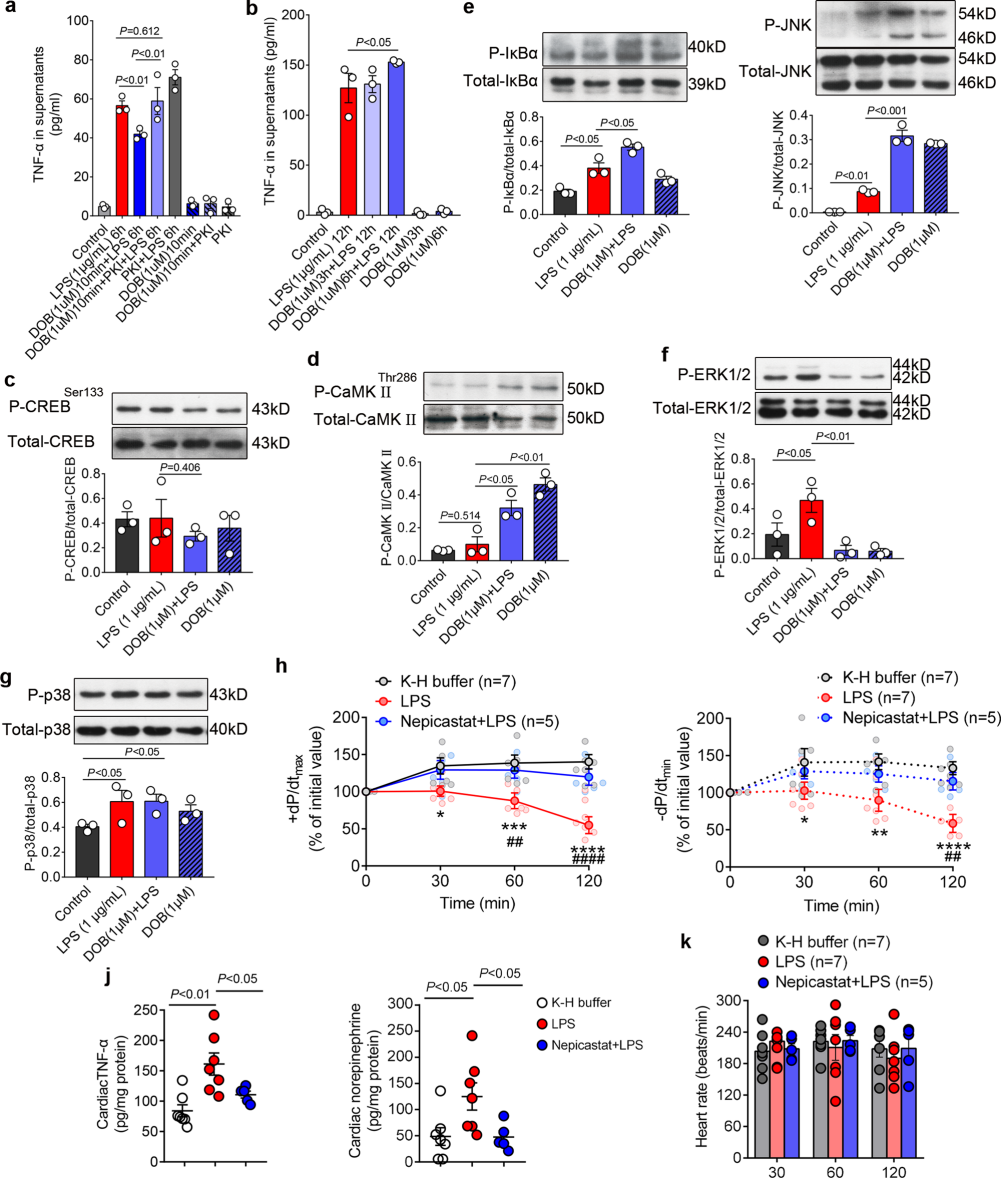
Dobutamine recapitulates the effects of ICA cell-derived NE, whereas blockade of ICA cell-derived NE prevents myocardial dysfunction induced by LPS. **a** TNF-α in supernatants of NRVM without ICA cell (NRVM ^ICA cell−^) treated with 1 µM dobutamine (DOB) for 10 min and/or 5 µM PKA inhibitor 14–22 amide (PKI) for 1 h prior to LPS 1 µg/mL for 6 h. **b** TNF-α in supernatants of NRVM ^ICA cell−^ treated with 1 µM DOB for 3 h or 6 h prior to 1 µg/mL LPS for 12 h. **c**–**g** Phosphorylation of CREB-Ser133, CaMKII-Thr286, IκBα, JNK, ERK1/2 and p38 in NRVM ^ICA cell−^ treated with 1 µM DOB for 6 h prior to 1 µg/mL LPS for 30 min. *n* = 36 neonatal rats, data represent three independent experiments (**a**–**g**). **h**, **i** and **k** Systolic (+d*P*/d*t*_max_), diastolic (−d*P*/d*t*_min_) function and heart rate of Langendorff-perfused adult rat hearts, LPS: 1.5 µg/mL, Nepicastat: Dopamine-β-hydroxylase (DBH) inhibitor, 15 µg/mL. **j** TNF-α and NE in myocardial homogenates from adult rat hearts Langendorff perfused for 120 min. *n* = 7 rats (K-H buffer), *n* = 7 rats (1.5 µg/mL LPS), *n* = 5 rats (15 µg/mL Nepicastat + LPS) in (**h**–**k**), each dot represents an adult rat, the solid dots on lines in (**h**) and (**i**) represent the mean of each group at each time point. Data are presented as mean ±  SEM. **a**–**g** and **j** one-way ANOVA and Bonferroni multiple comparison test, **h**, **i** and **k** two-way repeated measures ANOVA and Tukey’s multiple comparisons test. *P < 0.05, **P < 0.01, ***P < 0.001, ****P < 0.0001 in comparison of LPS vs. K-H buffer, ##*P* < 0.01, #### *P* < 0.0001 in comparison of LPS vs. Nepicastat + LPS. *P*-values were assigned on the panels.

The aforementioned data suggest that the NE from ICA cells contribute to the activation of NF-κB and MAPKs through β1-AR-CaMKII signaling in cardiomyocytes. We next employed dobutamine (DOB), a selective β_1_-AR agonist analogous to NE^36^, to recapitulate the contributions of ICA cells. We cultured NRVM without ICA cells (NRVM ^ICA cell−^) and then treated with DOB for different durations prior to LPS stimulation. Short-term (10-min) β_1_-AR stimulation with DOB reduced the LPS-induced TNF-α production in NRVM ^ICA cell−^, while PKA inhibitor 14–22 amide (PKI) increased LPS-induced TNF-α production and reversed the suppressive effect of DOB (Fig. 7a). Such observations are in agreement with published data suggesting that short-term β_1_-adrenergic stimulation elicits a rapid increase of cellular cAMP activating PKA^37^, which inhibits LPS-induced cardiac expression of TNF-α^38^. Of note, cardiomyocytes are perpetually regulated by intrinsic cardiac adrenergic activities. We thus prolonged the treatment time of DOB, and found that β_1_-AR stimulation for 6 h significantly increased the level of TNF-α in supernatants of LPS-treated NRVM ^ICA cell−^ (Fig. 7b). Similar to our observations regarding ICA cell-derived NE, DOB stimulation of NRVM ^ICA cell−^ for 6 h did not alter phosphorylation of CREB-Ser133 (Fig. 7c), but did dramatically enhance the level of phosphorylated-CaMKII^Thr286^ compared with control and LPS groups (Fig. 7d). The phosphorylation of CaMKII^Thr286^ in LPS-treated NRVM ^ICA cell−^ exhibited negligible differences compared with controls, showing that there was no NE production in NRVM ^ICA cell−^ culture due to the lack of ICA cells (Fig. 7d). Consistent with β_1_-AR blockade and CaMKII blockade in LPS-stimulated NRVM ^ICA cell+^, DOB treatment of NRVM ^ICA cell−^ for 6 h prior to LPS markedly increased phosphorylation of IκBα and JNK, whereas a decreased level of phosphorylated-ERK1/2 and comparable p38 phosphorylation when compared with the LPS alone group (Fig. 7e–g). Given that β_2_-AR activation suppresses p38 MAPK phosphorylation^39^, the unchanged p38 phosphorylation could be due in part to the mild β_2_-AR agonist activity of DOB^40^. Nonetheless, the data collectively shows DOB mimics ICA-cell derived NE that promotes TNF-α production in LPS-stimulated cardiomyocytes via β_1_-AR-CaMKII-NF-κB/MAPKs signaling cascades.

### Blockade of ICA cell-derived NE prevents LPS-induced myocardial dysfunction

The above mentioned data suggest a critical role of ICA cells in facilitating LPS-induced myocardial dysfunction. We, therefore, attempted to assess the influence of ICA cell-derived NE on cardiac function in vivo. Of note, circulating NE sourced from sympathetic nerves and adrenal medulla are significantly enhanced during sepsis^26^, however, the impact of ICA cell-derived NE on cardiac function is challenging to measure exclusively in vivo. Yet, the Langendorff perfusion system can eliminate the influence of circulating NE, and has proven invaluable and stalwart in studying pharmacological effects on myocardial function in the investigation of clinically relevant disease states such as heart failure^41,42^. It was thus utilized to investigate the impact of ICA cells on myocardial dysfunction induced by LPS in adult rats. The hearts in the LPS group exhibited significantly worse phenotypes in both systolic (+d*P*/d*t*_max_) and diastolic (−d*P*/d*t*_min_) functions than those in the K-H buffer group (control). Furthermore, blockade of ICA cell-derived NE biosynthesis using Nepicastat, an inhibitor of DBH, markedly rescued both systolic and diastolic functions compared to the LPS group (Fig. 7h, i). Nepicastat simultaneously abrogated the elevation of NE and TNF-α production in LPS-perfused hearts, consistent with changes in systolic and diastolic functions (Fig. 7j). The Langendorff-perfused hearts in different groups showed a negligible alteration in heart rate (Fig. 7k). These findings demonstrate that blockade of ICA cell-derived NE reduces LPS-induced cardiac TNF-α production and protects against LPS-induced myocardial dysfunction.

## Discussion

Catecholamines are irreplaceable in both developing and adult hearts. Targeted disruption in mice of the genes encoding catecholamine biosynthetic enzymes are embryonic lethal, likely due to cardiac failure^43,44^. The developing heart initially relies on ICA cells as the major source of catecholamines^45^. Published evidence also shows that ICA cells synthesize cardiac intrinsic catecholamines, occurring independently of cardiac sympathetic nerves, either in neonatal or adult hearts, thereby functioning as a critical and integral regulator in mammalian heart development, cardiac pathophysiology and post-heart transplantation support^14–17,19,21,46,47^. Stimulation of ICA cells enhances epinephrine release to reduce ischemia/reperfusion injury through the δ-opioid-regulated cardioprotective adrenopeptidergic co-signaling pathway^17,19,48^. Although ICA cell density shows negligible connection with reduced myocardial efficiency in failing myocardium^18^, irregular stimulation of ICA cells raises cardiac NE levels^49^. Considering the increase of cardiac NE levels and β_1_-AR activation aggravate sepsis-induced cardiomyocyte apoptosis and myocardial injury^8,11^, it is thus plausible that ICA cells could promote septic cardiomyopathy pathogenesis. Indeed, ICA cells are not separated from cardiomyocytes in most of the current cardiac models using primary neonatal rat or mouse ventricular myocytes (NRVM or NMVM)^50,51^. Our previous study established a method of superparamagnetic iron oxide particle (SIOP) to purify NRVM from ICA cells and demonstrated that the ICA cell-free NRVM showed lower calcium transient amplitude, longer time to begin autonomous beating and less expression of natriuretic peptide A (Nppa) and -B (Nppb) (markers of stress response) than those of NRVM mixed with ICA cells^24^. Moreover, we observed a reduced TNF-α production in ICA cell-free NRVM compared to unpurified cardiomyocytes in response to LPS stimulation^24^. Therefore, it is important to distinguish and evaluate the function of ICA cells in cardiac studies.

In the current study, we defined ICA cell-derived NE as a contributor to myocardial dysfunction via activation of β_1_-AR signaling in cardiomyocytes upon LPS challenge. Our data demonstrated ICA cells as a source of increased NE in LPS-treated neonatal and adult rat hearts. Although endothelial cells have been shown to produce catecholamines in response to ischemia^52^, the typical “cobblestone” monolayer pattern of morphology in endothelial cell culture largely differs from ICA cells^53^. In the Langendorff perfusion experiments, the unchanged NE levels in the perfusate from LPS-perfused hearts also indicate that the increased NE levels in the hearts were not from vascular endothelial cells. Utilization of a β_1_-AR blocker, CaMKII inhibitor, and β_1_-AR agonist showed that the NE from ICA cells can active cardiac β_1_-AR-CaMKII signaling, which crosstalks with NF-κB and MAPK pathways to promote TNF-α production and myocardial dysfunction during sepsis. This concept was further evidenced by the administration of Nepicastat inhibiting NE production in the ex vivo Langendorff model assessing septic heart function.

The early phase of sepsis has been characterized by high levels of circulating catecholamines, which boost the inflammatory response^26^, but the molecular mechanisms are multifactorial and not yet fully understood. TLR4, mediating recognition of LPS, has been well-known as the first-line sentinel of host defense against bacteria in innate immunity^26^. Previous studies show that TLR4 is present on immune cells and neurons^4,54^ which possess the capacity of catecholamine biosynthesis^25,55^. Whether TLR4 has functions in the regulation of catecholamine secretion remains elusive. We observed that TLR4 and its downstream MyD88/TRIF-AP-1 signaling pathway mediate LPS-induced biosynthesis of cellular NE through upregulation of NE-producing enzyme expression, assessed by the activation of TH-promoter. Consistently, a previous study showed that LPS continuously increased TH expression in mouse brains^56^, suggesting a promotive action to catecholamine biosynthesis for TLR4. Intriguingly, although the activation of TLR4 triggers cytokine release including TNF-α^26^, this is not the case in ICA cells despite TLR4 presence. This discrepancy could be due to the upregulation of *Nfkbia* and *Tnfaip6* expression that results in impaired p65 nuclear translocation in LPS-treated ICA cells. This concept fits well with published evidence that the increase of either *Nfkbia* or *Tnfaip6* expression suppresses NF-κB activation^30,31^. These findings hint towards a function of TLR4 signaling in hormone regulation during sepsis. In addition, the marked upregulations of chemokine family gene expression (i.e., *Cxc3cl1*, *ccl3*, *cxcl1*, *ccl2*, etc.) in LPS-treated ICA cells indicate that ICA cells may also contribute to cell migration involved in homeostatic and inflammatory processes.

PKA and CaMKII are two major downstream elements of β_1_-AR that is critical for the regulation of cardiac function in chronic heart failure^37,57^. In this study, we demonstrated an essential role for CaMKII in the pathogenesis of LPS-induced myocardial dysfunction. CaMKII activation was not observed in LPS-treated NRVM without the presence ICA cells in culture, whereas PKA activation showed no change. Recent evidence shows that CaMKII oxidation in response to myocardial infarction contributes to NF-κB-dependent inflammatory transcription in cardiomyocytes^58^. Moreover, it is also reported that oxidation-activated CaMKII has a causal role in the contractile dysfunction during sepsis^35^. Therefore, the present study demonstrates that ICA cell-derived NE is another factor in activating cardiomyocyte CaMKII via β_1_-AR to enhance LPS-induced cardiac inflammation, and β_1_-AR-CaMKII activation is the principal mechanism by which ICA cell-derived NE enhances cardiomyocyte NF-κB and MAPK signaling to promote LPS-induced cardiomyocyte TNF-α production.

**Fig. 8:**
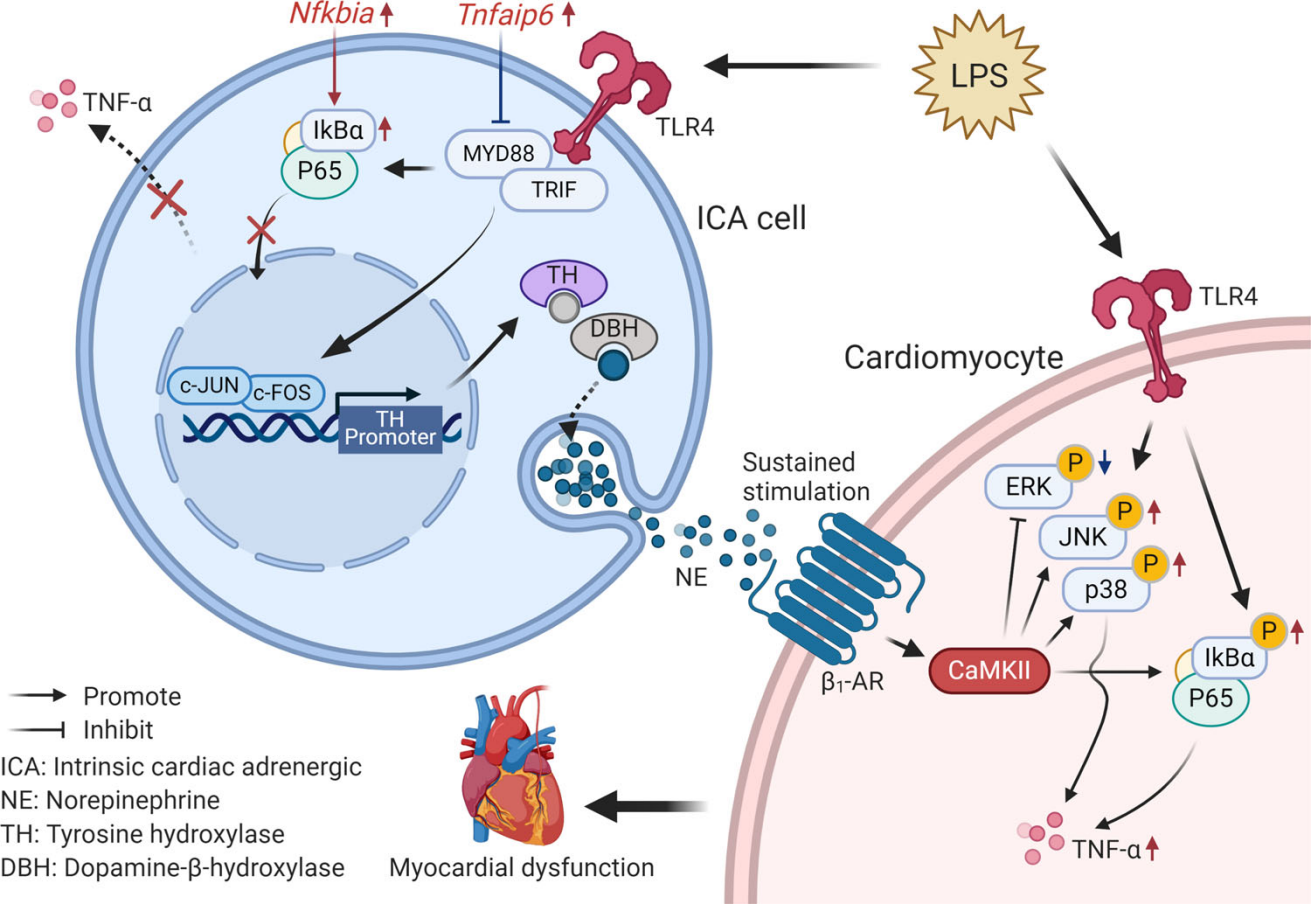
Schematic of ICA cell contribution to septic cardiomyopathy. ICA cell produces no TNF-α due to the elevated *Nfkbia* and *Tnfaip6* expression. Increased NE release by LPS-TLR4 signaling activates β1-AR-CaMKII signaling in cardiomyocytes to regulate NF-κB and mitogen-activated protein kinase pathways, subsequently aggravating myocardial TNF-α production and dysfunction.

In summary, we provide evidence for a pro-inflammatory role of ICA cells in LPS-induced myocardial dysfunction. Our data reveal that TLR4-MyD88/TRIF-AP-1 signaling is responsible for LPS-induced biosynthesis of NE that serves as a paracrine signal to aggravate LPS-induced myocardial TNF-α production and dysfunction via β_1_-AR-CaMKII signaling (Fig. 8). Considering the observation that blockade of ICA cell-derived NE prevents LPS-induced myocardial dysfunction in adult rat hearts, it seems plausible that ICA cells and CaMKII may be potential therapeutic targets in the fight against SIMD.

## Methods

### Animals and cell line

The experiments with animals were conducted in compliance with the Guide for the Care and Use of Laboratory Animals published by the US National Institutes of Health^59^ and approved by the Animal Care and Use Committee at Jinan University. Neonatal (1–3 days) and adult (8–10 weeks) Sprague-Dawley rats were obtained from the laboratory animal center of Southern Medical University (Guangzhou, China). TLR4-deficient mice (*Tlr4*^*Lps-del*^, strain: C57BL/10ScNJNju) were purchased from Nanjing Biomedical Research Institute of Nanjing University. For neonatal rats and mice, both male and female were used. For adult rats, male were used due to the impact of sex on septic cardiomyopathy^60^ and the differences in TNF-α levels and vascular reactivity of female following the administration of endotoxin^61^. HEK293/hTLR4-HA cells (InvivoGen) was a gift from Guang Yang (Jinan University).

### Inhibitors

α_1_-AR antagonist: Prazosin (Sigma-Aldrich#7791); α_2_-AR antagonist: Yohimbine (Sigma-Aldrich #Y3125); α_2A_-AR antagonist: BRL 44408 (Sigma-Aldrich #B4559); β_1_-AR antagonist: CGP20712A (Sigma-Aldrich #C231); β_2_-AR antagonist: ICI-118 551 (Sigma-Aldrich #I127); β_3_-AR antagonist: SR59230A (Sigma-Aldrich #S8688); TLR4 inhibitor: Viper peptide (Novus#NBP2-226244); CaMKII inhibitor: KN-93 Phosphate (Selleckchem #S7423); DBH inhibitor: Nepicastat (MCE MedChem Express#HY-13289); PKA Inhibitor: 14-22 Amide (Calbiochem® #476485).

### Isolation of primary NRVM and ICA cells

Co-culture of NRVM and ICA cells (NRVM ^ICA cell+^), NRVM without ICA cell (NRVM ^ICA cell−^) and ICA cells were isolated and purified using the published methods^24^. Briefly, the neonatal Sprague-Dawley rats (1–3 days) were deeply anesthetized by inhalation of CO_2_, and then the hearts were excised and transferred to pre-cold PBS. (1) NRVM ^ICA cell+^ isolation: the heart tissues were digested 5–6 times using 0.125% trypsin without EDTA (pH 7.30–7.40) to harvest NRVM ^ICA cell+^, and cultured in complete DMEM (10% FBS, 0.1 mM HEPES and 100U/ml Penicillin-Streptomycin) for downstream use (Fig. S1A). (2) NRVM ^ICA cell−^ purification using superparamagnetic iron oxide particles (SIOP) (BioMag#BM547): NRVM ^ICA cell+^ suspensions were centrifuged at 800 rpm, 4 °C for 7 min. The pellets were suspended in the pre-cold SIOP solution (40uL SIOP: 4 ml PBS), and then applied magnet (Life technologies™) to the side of the tube for 5-10 min to isolate NRVM ^ICA cell−^, which contained no ICA cells. The supernatant containing NRVM were centrifuged and the NRVM pellets were suspended in complete DMEM for culture. (3) ICA cell isolation: the SIOP binding ICA cells obtained from the last step were washed with ice-cold PBS, centrifuged, and suspended in complete DMEM for further use.

### Langendorff perfusion

Myocardial functions of the adult rats were measured using a Langendorff perfusion system as we described previously^62^. Briefly, the Sprague-Dawley rats (8–10 weeks) were heparinized (i.p. injection heparin, 2000 U) for 15 min, and then deeply anesthetized with isoflurane inhalation (3% isoflurane in 100% oxygen at a flow rate of 1 L/min) using a face mask. The hearts were isolated and the aortas were retrograde set up to a Langendorff perfusion apparatus (Radnoti Langendorff system#120102EZ) with a recirculating mode (volume of 50 mL) to perfuse at 10 mL/min with Krebs-Henseleit buffer (bubbled with 95% O_2_ and 5% CO_2_ gas mixture and maintained at 37 °C). Rat hearts were divided into groups of K-H buffer (control) or LPS (Sigma-Aldrich, #L2880, Escherichia coli, 055: B5, 1.5 µg/mL). The hearts were then perfused for 140 min with K-H buffer or LPS (1.5 µg/mL) using the recirculating mode, respectively. Perfusion fluid was collected at different time points, and heart tissues were harvested at the end of perfusion. In separate experiments, adult rat hearts were arranged into groups of K-H buffer (control), LPS or LPS + Nepicastat. After a 30-min equilibration perfusion in K-H buffer, a mixture of LPS (1.5 µg/mL) or/and Nepicastat (a selective DBH inhibitor^63^, 15 µg/mL) were perfused for 2 h. The physiological parameters of hearts were continuously recorded. The perfusate and left ventricular tissues were harvested for TNF-α and NE concentration determination as well as immunofluorescence staining (Fig. S1F).

### 10X genomics single-cell RNA-sequencing analysis

The strategy for single-cell RNA sequencing analysis of cardiac cells is shown in Fig. 5b. The neonatal Sprague-Dawley rats (1–3 days) were deeply anesthetized with CO_2_ and the hearts were excised and washed in cold PBS to remove the blood. Heart tissues were minced into ~1 mm^3^ masses and digested using 0.125% trypsin. (1) Cells were prepared using a modified Percoll gradient procedure described previously to enrich ICA cells^64^, of which details are described in Supplementary material. Immuno-staining of the cells was performed to examine the existence of ICA cells prior to single-cell RNA sequencing (Fig. S6A). (2) 10X genomics single-cell RNA sequencing: the cells were collected after treatment and analyzed to determine the viability of more than 90% by a cell count system. Individual samples were then loaded on the 10X Genomics Chromium System. Cells counted in the system were around 1.1–1.2 × 10^4^ per group (Fig. S6B). The libraries were prepared following 10X Genomics protocols and sequenced under standard procedure, followed by cell lysis and barcoded reverse transcription of RNA. The library construction and sequencing as well as computational analysis of data were performed at the Guangzhou Saliai Stem cell science and technology company. Single-cell gene expression was visualized in a two-dimensional projection with *t*-distributed stochastic neighbor embedding (t-SNE) map, in which all cells were grouped into 10 clusters (distinguished by their colors), and non-linear dimensional reduction was used (Fig. 5d)^65^. Analyses of batch effect correction and Differentially expressed genes (DEGs) were performed using the Seurat (version: 2.3.4) function, RunCCA and FindClusters; Resolution for granularity: 0.5; Differential expression test: Wilcoxon rank sum test; avg_logFC =  log(mean(group1)/mean(group2)); adjusted *p*_value: Bonferroni Correction; min.pct ≥10%; avg_logFC ≥ 0.1.

### Immunofluorescence staining

The immunofluorescence staining (IF) was performed according to our previous publication with minor modification^22^. Briefly, cells or tissue slices were fixed with 4% paraformaldehyde for 15 min followed by two washes with cold PBS and permeabilized with 0.25% Triton X-100 for 10 min. The specimens were then blocked at room temperature (RT) for 1 h and incubated with primary antibodies at 4 °C overnight. On the second day, the specimens were washed three times with cold PBS and then incubated in the dark with secondary antibodies at RT for 1 h, and then washed twice with cold PBS, followed by counterstaining with DAPI solution in the dark at RT for 10 min. The specimens were observed using a laser-scanning confocal microscope (Leica TCS SP8 X). Details of buffer preparation and antibody dilution are listed in Table S2.

### ELISA, Western blotting, and Quantitative RT-PCR assay

NE and TNF-α concentration in heart tissues, perfusate, and cell supernatants were determined using the NE research enzyme-linked immunosorbent assay (ELISA) kit (ALPCO#17-NORHU-E01-RES) and the TNF-α Quantikine ELISA kit (R&D System#RTA00), respectively. The western blotting assays of proteins were performed using standard protocol^22^. Antibodies for western blotting assays are available in the Supplementary Methods. Quantitative RT-PCR assay of relative mRNA expression was analyzed using standard real-time PCR protocol according to TAKARA qPCR kits. In brief, total RNA was reverse transcribed using a PrimeScript^TM^ RT Reagent Kit (TAKARA#RR047A). Real-time PCR were performed with the SYBR Premix Ex Taq II (TAKARA#RR820A) in a LightCycler480 real-time PCR system. Primer sequences for genes are shown in Table S3.

### Plasmids, molecular cloning, and reporter analysis

The pGL3-Basic Luciferase Reporter vector (Promega), pcDNA3.1 plasmid, plasmids encoding human TRIF and MyD88, RLTK-Luci, and AP-1-Luci reporters were a gift from Fuping You (Peking University)^66,67^. For reporter assays, a recombinant pGL3-Rat-TH-Luci reporter was designed and cloned (Fig. S4B–D), and then verified by sequencing the relevant region (Supplementary Data 2, Fig. S4E, F). The EGFP plasmid was used as transfection efficiency control (Fig. S4G). HEK293/hTLR4-HA cells seeded in 24-well plates were transfected with 50 ng of the luciferase reporter together with a total of 300 ng various expression plasmids or control plasmids. Quantification of dual-luciferase activity was performed using a GALEN dual-luciferase assay kit (GALEN#GN201).

### RNA interference

RNA interference was designed to disrupt AP-1 binding in the rat TH promoter region (Fig. S5A–C). c-Jun siRNA, c-Fos siRNA (Sequences refer to Table S4), and control siRNA transfection were processed using Lipofectamine^®^3000 Reagent (Invitrogen™#L3000001) and Opti-MEM (Gibco#31985-070) under a standard protocol.

### Isolation of rat peritoneal macrophages

Rat peritoneal macrophages were isolated using a published method^68^. Adult rats (250–300 g) were euthanized with CO_2_ plus cervical dislocation, and then the abdomen was soaked with 70% alcohol followed by making a small incision along the midline. 10 ml of DMEM were injected into each rat peritoneal cavity and gently massaged. About ~8 ml of fluid was aspirated from the peritoneum per rat. The peritoneal cells were collected by centrifuging for 10 min, 400 × *g* at 4 °C, and cultured for downstream use.

### Statistics and reproducibility

All data were analyzed for statistical significance with SPSS 13.0 software (SPSS Inc., Chicago, IL, USA). Two-tailed independent Student’s *t-test* was used for two groups comparison. More than two groups were compared using one-way analysis of variance (ANOVA) followed by Bonferroni multiple comparison test for normally distributed and equal variance data, or nonparametric Kruskal–Wallis test and Dunn’s method of comparison for non-normal distributions. Data are presented as mean ± SEM, where *n* is the number of animals. *P* < 0.05 was considered statistically significant. ^∗^*P* < 0.05, ^∗∗^*P* < 0.01, ^∗∗∗^*P* < 0.001. Statistical details, including sample sizes and experiment repeats, are reported in the figures and figure legends.

### Reporting summary

Further information on research design is available in the Nature Research Reporting Summary linked to this article.

## Supplementary information

Supplementary Information

Description of Additional Supplementary Files

Supplementary Data 1

Supplementary Data 2

Reporting Summary

## Data Availability

The data generated in this study are included in the paper and Supplementary files, and available from the corresponding author upon reasonable request. The single-cell RNA-seq data have been deposited in GEO (GSE189964). The uncropped gel and images for western blots are provided in the Supplementary Information. The source data behind all graphs and charts in the main manuscript are included in the Supplementary Data 1 file. The sequence of the recombinant pGL3-Rat-TH-Luci reporter plasmid is in the Supplementary Data 2 file.
