# Supplementary material for: Intrinsic cardiac adrenergic cells contribute to LPS-induced myocardial dysfunction
Source: Commun Biol. 2022 Jan 25;5:96. doi: 10.1038/s42003-022-03007-6 (PMC8789803; doi:10.1038/s42003-022-03007-6)
Supplement: Supplementary file 2 — Description of Additional Supplementary Files [file 42003_2022_3007_MOESM2_ESM.pdf]

Description of Additional Supplementary Files

File name: Supplementary Data 1

Description: Source data behind all graphs and charts.

File name: Supplementary Data 2

Description: Sequence of pGL3-Rat-TH promoter luciferase reporter plasmid.
