# Supplementary material for: Intrinsic cardiac adrenergic cells contribute to LPS-induced myocardial dysfunction
Source: Commun Biol. 2022 Jan 25;5:96. doi: 10.1038/s42003-022-03007-6 (PMC8789803; doi:10.1038/s42003-022-03007-6)
Supplement: Supplementary file 5 — Reporting Summary [file 42003_2022_3007_MOESM5_ESM.pdf]

nature research

Corresponding author(s): Huadong Wang
Last updated by author(s): Nov 25, 2021

Reporting Summary

Nature Research wishes to improve the reproducibility of the work that we publish. This form provides structure for consistency and transparency in reporting. For further information on Nature Research policies, see our Editorial Policies and the Editorial Policy Checklist.

Statistics

For all statistical analyses, confirm that the following items are present in the figure legend, table legend, main text, or Methods section.

n/a	Confirmed	
☐	☒	The exact sample size (*n*) for each experimental group/condition, given as a discrete number and unit of measurement
☐	☒	A statement on whether measurements were taken from distinct samples or whether the same sample was measured repeatedly
☐	☒	The statistical test(s) used AND whether they are one- or two-sided<br>*Only common tests should be described solely by name; describe more complex techniques in the Methods section.*
☐	☒	A description of all covariates tested
☒	☐	A description of any assumptions or corrections, such as tests of normality and adjustment for multiple comparisons
☐	☒	A full description of the statistical parameters including central tendency (e.g. means) or other basic estimates (e.g. regression coefficient) AND variation (e.g. standard deviation) or associated estimates of uncertainty (e.g. confidence intervals)
☒	☐	For null hypothesis testing, the test statistic (e.g. *F*, *t*, *r*) with confidence intervals, effect sizes, degrees of freedom and *P* value noted<br>*Give P values as exact values whenever suitable.*
☒	☐	For Bayesian analysis, information on the choice of priors and Markov chain Monte Carlo settings
☒	☐	For hierarchical and complex designs, identification of the appropriate level for tests and full reporting of outcomes
☒	☐	Estimates of effect sizes (e.g. Cohen's *d*, Pearson's *r*), indicating how they were calculated

Our web collection on statistics for biologists contains articles on many of the points above.

Software and code

Policy information about availability of computer code

Data collection	N/A
Data analysis	N/A

For manuscripts utilizing custom algorithms or software that are central to the research but not yet described in published literature, software must be made available to editors and reviewers. We strongly encourage code deposition in a community repository (e.g. GitHub). See the Nature Research guidelines for submitting code & software for further information.

Data

Policy information about availability of data

All manuscripts must include a data availability statement. This statement should provide the following information, where applicable:

- Accession codes, unique identifiers, or web links for publicly available datasets
- A list of figures that have associated raw data
- A description of any restrictions on data availability

Fig.1, 2, 3, 4, 5, 6, 7 have associated source data. The datasets generated during and/or analysed during the current study are available from the corresponding author on reasonable request.

Field-specific reporting

Life sciences study design

All studies must disclose on these points even when the disclosure is negative.

Sample size	The sample size chosen for our animal and cell experiments in this study was estimated based on our prior experience of performing similar sets of experiments and power analysis calculations. (https://isogenic.info/html/power_analysis.html)
Data exclusions	No data were excluded from the analyses.
Replication	3-6 independent reproducible experiments were performed. At least 3 biological replicates were included for each experiment/each condition where a statistical analysis was performed.
Randomization	All animal or cell results were included and no method of randomization was applied.
Blinding	Some IF slides were stained in a blind manner.

Reporting for specific materials, systems and methods

We require information from authors about some types of materials, experimental systems and methods used in many studies. Here, indicate whether each material, system or method listed is relevant to your study. If you are not sure if a list item applies to your research, read the appropriate section before selecting a response.

Materials & experimental systems

n/a	Involved in the study
☐	☒ Antibodies
☐	☒ Eukaryotic cell lines
☒	☐ Palaeontology and archaeology
☐	☒ Animals and other organisms
☒	☐ Human research participants
☒	☐ Clinical data
☒	☐ Dual use research of concern

Methods

n/a	Involved in the study
☒	☐ ChIP-seq
☒	☐ Flow cytometry
☒	☐ MRI-based neuroimaging

Antibodies

Antibodies used	TH (Abcam#ab112), TLR4 (Abcam#ab22048), DBH (Sigma Aldrich#SAB2701977), P-p65 (CST#3033), P65 (CST#4764), extracellular signalregulated kinase (ERK) 1/2 (CST#4695), P-ERK1/2 (CST#4370), c-jun NH2-terminal kinase (JNK)1/2 (CST#9252), P-JNK1/2 (CST#4668S), p38MAPK (CST#9212S), P-p38 (CST#4511), IκBα (CST#4812S), P-IκBα (CST#9246), glyceraldehyde-3-phosphate dehydrogenase (GAPDH) (CST#2118), c-Fos (CST#2250), c-Jun (CST#9165), CaMKII (CST#3362), P-CaMKIIThr286 (CST#12716T), P-CREBSer133 (Affinity#AF3189), CREB (Affinity#AF6188).
Validation	These antibodies have been validated by other publications. We also validated some of them with specific gene knockout cell lysates.

Eukaryotic cell lines

Policy information about cell lines

Cell line source(s)	Human embryonic kidney 293 cells transformed with TLR4, 293/hTLR4-HA cells (InvivoGen#293-htlr4ha)
Authentication	This cell line are from and has been authenticated by InvivoGen.
Mycoplasma contamination	To ensure the cell culture are free of mycoplasma, we regularly treated cells with Mycozap (#Lonza)
Commonly misidentified lines (See ICLAC register)	This cell line is not listed in the database of commonly misidentified cell lines maintained by ICLAC.

Animals and other organisms

Policy information about studies involving animals; ARRIVE guidelines recommended for reporting animal research

Laboratory animals	The neonatal (1-3 days old) and adult (6-8 weeks) Sprague-Dawley rats were obtained from the laboratory animal center of Southern Medical University (Guangzhou, China). TLR4-deficient mice (Tlr4Lps-del, strain: C57BL/10ScNJNju) were purchased from Nanjing Biomedical Research Institute of Nanjing University.
Wild animals	N/A

Field-collected samples	N/A
Ethics oversight	All experiments with animals were conducted in compliance with the Guide for the Care and Use of Laboratory Animals published by the US National Institutes of Health and approved by the Animal Care and Use Committee at Jinan University (LAECJU20180225016).

Note that full information on the approval of the study protocol must also be provided in the manuscript.
